# Influence of the Poison of Northern Rattle-Snakes on Plants

**Published:** 1851-10

**Authors:** J. H. Salisbury

**Affiliations:** Chemist to New York State Agricultural Society


*Influence of the Poison of the Northern Rattle-Snake (Crotalus Durissus) on Plants.* By J. H. Salisbury, M. D., Chemist to New York State Agricultural Society.—On the 18th of June, 1851, a large female rattlesnake—which had been caged in the New York State Cabinet of Natural History, died. On dissection, its stomach and intestinal canal were found entirely empty, as much so as if they had been scoured out with soap suds. The sack in which the poison is emptied was laid open, and the virulent matter, (of which there was but little,) carefully removed and placed in a porcelain capsule. About five minutes after its removal, four young shoots of the lilac, (syringa vulgaris); a small horse chestnut, (æsculus hippocastanum), of one year's growth; a corn plant, (zea maix); a sunflower plant, (helianthus americus); and a wild cucumber vine, were vaccinated with it. The vaccination was performed by the dipping the point of a penknife into the virulent matter, and then inserting it into the plant, just beneath the inner bark. No visible effect, in either case, of the influence of the poison was perceptible, till about sixty hours after it had been inserted. Soon after this, the leaves above the wound, in each case, began to wilt. The bark in the vicinity of the incision exhibited scarcely a perceptible change; in fact, it would have been difficult to have found the points, if they had not been marked, where the poison was inserted.

Ninety-six hours after the operation, nearly all the leaf-blades, in each of the plants, above the wounded part, were wilted and quite dead. On the fifth day, the petioles and bark, above the incisions, began to lose their freshness, and on the sixth day they were considerably withered. On the seventh day they were about as they were on the sixth. On the tenth day they began to show slight signs of recovery. On the fifteenth day, new, but sickly appearing leaves began to show themselves on the lilacs, and the other plants began to present slight signs of recovery in the same way. Neither of the plants were entirely destroyed. It was interesting to mark the progressive influence of the poison. The first indication of the derangement of the healthy functions of the plants was observed in the leaves. These began to wilt and die at their edges and apices, and this death gradually and uniformly advanced on all sides towards the mid-rib and petiole, till the whole or nearly the entire leaf was destroyed.

It is an interesting fact in physiology that the plants first exhibited signs of death in the leaves situated on the side in which the incisions were made. This shows a less perfect system of anastomosing vessels than in the animal.

The facts naturally deducible from these experiments, are:—

That the effects of the poison of the rattlesnake upon plants and animals, when introduced into their circulation by a wound, are similar. It is stated on good authority that the poison of the snake can be taken into the stomach of the animal with impunity—its dangerous effects being only exhibited when introduced into the circulation, by a wound. It would be interesting to note its influence on the animal when applied externally to the skin, to see whether its deadly effects would be modified by the absorbents.

That it requires a much longer time for it to affect the plant than the animal. It should be stated, in order to show that animals were readily affected by the poison of the snake, that a short time previous to its death, a rat, bitten by it, died in about two hours.

That the effects were invariably exhibited on the part above the wound, and in no case affected the leaves below it. This was probably owing to the small quantity of the poison introduced in each instance.

That it invariably affected first the leaves on the side of the plant in which the incision was made.

That its influence was invariably first rendered visible on the edges and apices of the leaf blades. In this respect the effects of this poison on plants resembles strikingly, in its beginning and progress, the disease which has for the last few years affected so much the potato.—*Proceedings Am. Sci. Asso.*

